# Secondary prevention therapies following percutaneous coronary intervention or acute coronary syndrome in patients with diabetes mellitus

**DOI:** 10.3389/fcvm.2024.1436332

**Published:** 2024-11-22

**Authors:** Arnaud Planchat, Baris Gencer, Sophie Degrauwe, Yazan Musayeb, Marco Roffi, Juan F. Iglesias

**Affiliations:** ^1^ Department of Cardiology, Geneva University Hospitals, Geneva, Switzerland; ^2^ Department of Cardiology, Lausanne University Hospital, Lausanne, Switzerland

**Keywords:** diabetes mellitus, coronary artery disease, percutaneous coronary intervention, secondary prevention, antithrombotic therapy

## Abstract

Diabetes mellitus (DM) promotes atherosclerosis, leading to increased risk for cardiovascular morbidity and mortality. Diabetics represent a challenging subset of patients undergoing percutaneous coronary intervention (PCI) or who have experienced an acute coronary syndrome (ACS), a subset characterized by higher rates of recurrent ischemic events compared with non-diabetics. These events are caused by both patient-related accelerated atherosclerotic disease progression and worse stent-related adverse clinical outcomes translating into a higher risk for repeat revascularization. In addition, DM is paradoxically associated with an increased risk of major bleeding following PCI or an ACS. Secondary prevention therapies following PCI or an ACS in diabetic patients are therefore of paramount importance. This mini review focuses on the currently available evidence regarding short- and long-term secondary prevention treatments for diabetic patients undergoing PCI or who have experienced an ACS.

## Introduction

Diabetes mellitus (DM) is assuming the shape of a pandemic that is expected to affect 63 million individuals by 2045 and directly contribute to 6.7 million deaths worldwide (1). DM is a major risk factor for atherosclerosis (2), leading to multisite vascular involvement (3). Diabetics face a nearly twofold increased risk of major adverse cardiovascular events (MACE), including coronary artery disease (CAD), stroke, or cardiovascular (CV) death, independently from other conventional CV risk factors (4, 5). In terms of CAD, diabetes confers an equivalent risk to 15 years of aging (4).

CV disease is the leading cause of death among diabetic patients, responsible for about two-thirds of their mortality, with CAD accounting for nearly 40% of these deaths (6). DM is increasingly prevalent among patients undergoing coronary revascularization (7). Diabetics represent a challenging subset of patients undergoing percutaneous coronary intervention (PCI) or who have experienced an acute coronary syndrome (ACS), a subset characterized by higher rates of recurrent ischemic events compared with non-diabetics. These events are caused by both patient-related accelerated atherosclerotic disease progression and worse stent-related adverse outcomes translating into a higher risk for repeat revascularization (8). In this study, we review current evidence on available secondary prevention therapies for diabetic patients who underwent PCI or who experienced an ACS.

## Diabetes-associated atherothrombosis

Among diabetics, DM itself remains the main cause of accelerated atherosclerosis (8). Diabetes promotes atherogenesis and atherothrombosis through several mechanisms, including endothelial dysfunction, prothrombotic and proinflammatory states, and metabolic disturbances such as hyperglycemia, dyslipidemia, obesity, insulin resistance, and oxidative stress (9). Endothelial dysfunction is a key feature, mediated by hyperglycemia, increased free fatty acid production, altered lipoproteins, and insulin resistance (9). Diabetes is also characterized by platelet hyperreactivity (10) resulting from hyperglycemia, which induces the expression of multiple platelet activation receptors [glycoprotein (GP) Ib, GP IIb/IIIa, and P2Y_12_] and glycation of platelet surface proteins, leading to insulin resistance, reduced membrane fluidity, and higher thromboxane A2 production (11). Diabetes-associated oxidative stress increases the production of isoprostanes which, combined with thromboxane A2 (TxA_2_), induce platelet activation (11). Diabetes is also associated with accelerated platelet turnover that conveys a reduced response to antiplatelet agents (12). Additional metabolic conditions associated with diabetes (obesity, dyslipidemia, or systemic inflammation) may also increase thrombotic risk (13). Diabetes may also induce a prothrombotic state because of alterations in the coagulation-fibrinolytic system. Diabetics have increased levels of tissue factor, prothrombin factor VII, and fibrinogen which, combined with lower levels of anticoagulant proteins and impaired fibrinolytic activity (14), contribute to the formation of thrombi resistant to fibrinolysis (15). However, despite an enhanced prothrombotic environment, diabetics have a paradoxically increased risk of major bleeding events, particularly in the setting of potent antithrombotic agents (16).

## Short-term antithrombotic therapies following PCI in diabetic patients

Diabetes-induced atherothrombosis may impact clinical outcomes following PCI. Diabetic patients exhibit reduced sensitivity to thienopyridine-based P2Y_12_ inhibitors compared with non-diabetic individuals, but not to ticagrelor (17, 18), although the clinical significance of these findings remains unclear. Current treatment strategies aimed at reducing the risk of adverse thrombotic events following PCI in diabetic patients focus on the early introduction of therapies inhibiting platelet function.

### Aspirin

Aspirin is the mainstay of antithrombotic therapy for secondary prevention following PCI or an ACS (19, 20). Because of increased platelet turnover observed in diabetics and aspirin short half-life, platelet cyclooxygenase activity may recover during dosing intervals, thus limiting the duration of efficacious antiplatelet effects of low-dose aspirin in diabetics (12). Although based on pharmacodynamic studies a twice-daily low-dose aspirin regimen has been suggested to overcome inadequate TxA_2_ inhibition, no clinical application was found by researchers (12). Accordingly, current available data emanating from randomized clinical trials (RCTs) including a sizable proportion of diabetic patients showed no difference between low- and high-dose aspirin with regard to ischemic or major bleeding events with established atherosclerotic CV disease (21, 22) or in ACS (21). The ANDAMAN RCT (NCT02520921) is currently investigating the clinical benefits of a twice-daily, compared with a once-daily, aspirin administration in terms of the composite of all-cause death, myocardial infarction (MI), stroke, urgent coronary revascularization, and/or stent thrombosis (ST), or acute arterial thrombotic event at 18 months among 2,574 ACS patients with diabetes or a risk factor for non-optimal aspirin response planned for PCI.

### Dual antiplatelet therapy

Dual antiplatelet therapy (DAPT), combining low-dose aspirin with a P2Y_12_ receptor inhibitor, is the standard treatment to prevent ischemic events—in particular ST—following drug-eluting stent (DES) implantation (19). Initially, a DAPT duration of at least 12 months following PCI was recommended based on early studies with a first-generation DES (23). Following the introduction of a new-generation DES with improved biocompatibility, recent RCTs have explored the efficacy and safety of a shorter DAPT following PCI and demonstrated significant reductions in major bleeding events while maintaining similar efficacy with regard to MACE with a shorter DAPT (24). Furthermore, the advent of more potent P2Y_12_ receptor antagonists, ticagrelor and prasugrel, was associated with a significant reduction in MACE, although at the cost of a higher risk of major bleeding events, and became the standard of care for an ACS (25, 26). Current guidelines recommend DAPT with aspirin and clopidogrel for 6 months following PCI in patients with a chronic coronary syndrome (CCS), whereas DAPT, combining aspirin and preferably prasugrel or ticagrelor, is advocated for 12 months in patients with an ACS following PCI (19). For high-bleeding-risk patients undergoing PCI, a reduction of DAPT duration to 1–3 and 6 months for patients with a CCS and ACS, respectively, (19) is recommended.

Among patients with a CCS, clopidogrel is recommended in combination with low-dose aspirin following PCI, whereas prasugrel or ticagrelor may be considered only in specific high-risk PCI settings, such as patients with prior ST or those undergoing left main revascularization (19). The HOST-EXAM trial, which included a predominantly East Asian cohort (*n* = 5,530, 34% diabetics), and which compared clopidogrel monotherapy with aspirin after a period of DAPT, found a reduction in the composite of death, MI, stroke, and significant bleeding with clopidogrel at a follow-up of 24 months (27). The treatment effect was consistent after 5–8 years among diabetic patients in subgroup analyses (27, 28). However, the generalizability of these results, especially outside East Asian populations, remains uncertain (29).

Several clinical trials have assessed different antiplatelet therapies in patients with ACS to balance the benefits of reducing MACE with the risks of major bleeding. The CURE trial (*n* = 12,562, 23% diabetics) demonstrated the incremental benefits of adding clopidogrel to aspirin on reducing MACE in patients with ACS, at the expense of an increased major bleeding risk (30). Those results were consistent across diabetic and non-diabetic patients (30). Higher doses of clopidogrel did not offer additional benefits and only increased the risk of major bleeding events (21).

In the TRITON-TIMI 38 study (*n* = 13,698, 23% diabetics), it was found that prasugrel reduced MACE rates but increased the risk of major bleeding events (25). Diabetic patients seemed to benefit more from prasugrel than from clopidogrel, with similar major bleeding rates (25). In the PLATO trial, ticagrelor demonstrated lower MACE rates compared with clopidogrel, without significantly increasing major bleeding events (26). A subgroup analysis of diabetic patients showed consistent benefits with ticagrelor, including reduced all-cause mortality and ST without increasing the risk of major bleeding events (16). In the ISAR-REACT 5 trial (*n* = 4,018, 22% diabetics), prasugrel was compared with ticagrelor in addition to low-dose aspirin in patients with ACS undergoing PCI (31). At 1 year, patients treated with ticagrelor had higher rates of the primary composite endpoint of CV death, MI, or stroke compared with those treated with prasugrel, without any significant differences in major bleeding events (31). The rates of the primary composite endpoint did not significantly differ between the two treatments for diabetic patients (32). Major bleeding rates were similar in both diabetic and non-diabetic subjects, regardless of the antiplatelet agent used (32).

### Single antiplatelet therapy

Single antiplatelet therapy (SAPT) with a P2Y_12_ receptor inhibitor has recently emerged as an attractive de-escalation strategy to limit the risk of bleeding irremediably associated with DAPT (24). Several RCTs (Table 1) have investigated the efficacy and safety profiles of a short (≤3 months) DAPT strategy, followed by a P2Y_12_ inhibitor SAPT, compared with a standard DAPT among patients with a CCS and ACS.

**Table 1:** Summary of major randomized clinical trials and subgroup analyses comparing single antiplatelet therapy with standard double antiplatelet therapy in patients with diabetes after PCI.

Study	Year	Patients (diabetic %)	Intervention	Follow-up	Outcomes
GLOBAL LEADERS (+ prespecified analysis and *post hoc* analysis)	2018	15,968 patients (diabetics, 26%; insulin-treated, 8%)	• Aspirin and ticagrelor for 1 month followed by ticagrelor monotherapy for 23 months • Standard DAPT with aspirin and either clopidogrel in patients with CCS or ticagrelor in patients with ACS for 12 months, followed by aspirin monotherapy for 12 months	2 years	• Ticagrelor monotherapy strategy fell short of demonstrating superiority with respect to the primary composite ischemic endpoint of all-cause death, or non-fatal centrally adjudicated new Q-wave MI despite an apparent trend favoring the experimental strategy in the intention-to-treat analysis (3.8% vs. 4.4%; RR, 0.87; 95% CI, 0.75–1.01; *p* = 0.07) • No significant differences in the rates of major bleeding (2.0% vs 2.1%; RR, 0.97; 95% CI, 0.78–1.20; *p* = 0.77) • In a prespecified subgroup analysis (4,038 diabetic patients), no significant treatment effect was found with respect to the primary combined ischemic endpoint and major bleeding outcomes at 2 years in patients with and without diabetes • In a *post hoc* analysis including 838 patients with concomitant diabetes and chronic kidney disease, it was found that ticagrelor monotherapy did not significantly reduce the rates of all-cause death, or new Q-wave MI (8.4% vs. 10.7%; HR, 0.75; 95% CI, 0.47–1.18, *p* = 0.09), and major bleeding complications (4.2% vs. 4.6%; HR, 0.86; 95% CI, 0.45–1.64, *p* = 0.59) compared with a standard DAPT regimen, but it was associated with lower rates of the patient-oriented composite endpoint of all-cause death, any stroke, site-reported MI, or any revascularization (20.6% vs. 25.9%; HR, 0.74; 95% CI, 0.55–0.99, *p* = 0.043) and net adverse clinical events (composite of the patient-oriented composite endpoint, or major bleeding events) (22.7% vs. 28.3%; HR, 0.75; 95% CI, 0.56–0.99, *p* = 0.044), a difference mainly driven by a lower risk of any revascularization, compared with the reference DAPT regimen
TWILIGHT (+ prespecified analysis)	2019	7,119 patients (diabetics, 37%; insulin-treated, 10%) at a high risk of ischemic or bleeding events after PCI with newer-generation DES and completion of a 3-month course of DAPT with aspirin and ticagrelor without major bleeding or ischemic events	Aspirin or matching placebo, along with the continuation of open-label ticagrelor treatment, for an additional 12 months (1:1 ratio)	1 year	• Ticagrelor monotherapy was associated with a significant reduction in clinically relevant major bleeding (4.0% vs. 7.1%; HR, 0.56; 95% CI, 0.45–0.68; *p* < 0.001) compared with standard DAPT with aspirin and ticagrelor • No significant differences in the rates of the composite ischemic endpoint of all-cause death, MI, or stroke at 1 year between the two treatment strategies, thus meeting the prespecified non-inferiority hypothesis (HR, 0.99; 95% CI, 0.78–1.25; *p* < 0.001) • In a prespecified analysis including 2,620 diabetic patients, it was found that the effect of ticagrelor monotherapy in reducing the risk of clinically relevant bleeding was consistent among patients with (4.5% vs. 6.7%; HR, 0.65; 95% CI, 0.47–0.91; *p* = 0.01) or without (3.8% vs. 7.3%; HR, 0.50; 95% CI, 0.39–0.66; *p* < 0.001) diabetes undergoing PCI (*p* for interaction = 0.23) • Significant treatment-by-diabetic status interaction with respect to the occurrence of the composite ischemic endpoint favoring a ticagrelor monotherapy strategy over standard DAPT among diabetic (4.6% vs. 5.9%; HR, 0.77; 95% CI, 0.55–1.09; *p* = 0.14) but not among non-diabetic (3.5% vs. 2.8%; HR, 1.24; 95% CI, 0.89–1.73; *p* = 0.21), subjects (*p* for interaction = 0.05)
TICO	2020	3,056 patients with ACS (diabetics, 27%; ST-elevation MI, 36%)	• Ticagrelor monotherapy after 3-month DAPT • Ticagrelor-based 12-month DAPT following PCI with newer-generation DES	1 year	• Ticagrelor SAPT after a 3-month DAPT was associated with a modest, but significant, reduction in the primary composite endpoint of major bleeding and MACE (3.9% vs. 5.9%; HR, 0.66; 95% CI, 0.48–0.92; *p* = 0.01), a difference mainly driven by a lower risk for major bleeding with ticagrelor SAPT (1.7% vs. 3.0%; HR, 0.56; 95% CI, 0.34–0.91; *p* = 0.02) • No significant differences were found with respect to the composite ischemic outcome of all-cause death, MI, stent thrombosis, stroke, or target vessel revascularization (TVR) (2.3% vs. 3.4%; HR, 0.69; 95% CI, 0.45–1.06; *p* = 0.09) between the two treatment strategies • Treatment effect consistent among patients with (*n* = 835) and without diabetes (*p* for interaction = 0.65) (33)
HOST-EXAM	2021	5,530 Southeast Asian patients (diabetics, 34%), on DAPT without adverse clinical events for 6–18 months after PCI with DES	• Low-dose aspirin (100 mg) • Clopidogrel (75 mg) monotherapy	2 years	• Clopidogrel monotherapy was found superior to aspirin monotherapy with respect to the combined endpoint of all-cause death, MI, stroke, readmission due to ACS, or major bleeding events (5.7% vs. 7.7%; HR, 0.73; 95% CI, 0.59–0.90; *p* = 0.0035) compared with aspirin monotherapy • Treatment effect was consistent among patients with diabetes (*p* for interaction = 0.65)
STOPDAPT-2 ACS	2022	4,169 patients with ACS (diabetics, 30%; ST-elevation MI, 56%)	• 1–2 months of DAPT followed by a less potent P2Y12 receptor inhibitor SAPT using clopidogrel • 12-month DAPT with aspirin and clopidogrel following PCI with newer-generation DES	1 year	• Clopidogrel SAPT after 1–2 months of DAPT fell short of demonstrating non-inferiority compared with standard 12-month DAPT with respect to the primary composite endpoint of CV death, MI, any stroke, definite stent thrombosis, and major or minor bleeding events (3.2% vs. 2.8%; HR, 1.14; 95% CI, 0.80–1.62; *p* for non-inferiority = 0.06), with a numerical increase in CV events (2.8% vs. 1.9%; HR, 1.50; 95% CI, 0.99–2.26) despite significant reductions in major bleeding events (0.5% vs. 1.2%; HR, 0.46; 95% CI, 0.23–0.94) • Treatment effect consistent among patients with (*n* = 1,229) and without diabetes
STOPDAPT-3	2023	6,002 patients with ACS or high bleeding risk just before PCI (diabetics, 40%, ACS, 75%)	• Prasugrel (3.75 mg/day) monotherapy • DAPT with aspirin (81–100 mg/day) and prasugrel (3.75 mg/day)	1 month	• The no-aspirin group of patients was not superior to the DAPT group of patients in terms of coprimary bleeding endpoint (4.47% and 4.71%; hazard ratio, 0.95, 95% CI, 0.75–1.20; *p*_superiority_ = 0.66) • The no-aspirin group was non-inferior to the DAPT group in terms of the coprimary cardiovascular endpoint (4.12% and 3.69%; hazard ratio, 1.12, 95% CI, 0.87–1.45; *p*_non-inferiority_ = 0.01) • There was no difference in net adverse clinical outcomes and each component of coprimary cardiovascular endpoint • There was an excess of any unplanned coronary revascularization (1.05% and 0.57%; HR, 1.83; 95% CI, 1.01–3.30) and subacute definite or probable stent thrombosis (0.58% and 0.17%; HR, 3.40, 95% CI, 1.26–9.23) in the no-aspirin group compared with the DAPT group • Treatment effect was consistent among patients with diabetes (5.08% vs. 4.98%; HR, 1.02; 95% CI, 0.72–1.46; *p* for interaction = 0.44)
T-PASS	2023	2,850 patients with ACS (diabetics, 30%; ST-elevation MI, 40%)	• Ticagrelor monotherapy (90 mg twice daily) after <1 month of DAPT • 12 months of ticagrelor-based DAPT	1 year	• Aspirin was discontinued at a median of 16 days (interquartile range, 12–25 days) in the group receiving ticagrelor monotherapy after <1 month of DAPT • Stopping aspirin within 1 month (median of 16 days) for ticagrelor SAPT is both non-inferior and superior to 12-month DAPT with regard to the 1-year composite outcome of death, MI, stent thrombosis, stroke, and major bleeding (2.5% vs. 5.2%, HR, 0.54; 95% CI, 0.37–0.80; *p* < 0.001 for non-inferiority; *p* = 0.002 for superiority) • The occurrence of major bleeding events was significantly lower in the group receiving ticagrelor monotherapy after <1 month of DAPT compared with the 12-month DAPT group (1.2% vs. 3.4%; HR, 0.35; 95% CI, 0.20–0.6]; *p* < 0.001) • Significant treatment-by-diabetic status interaction with respect to the occurrence of the composite ischemic endpoint favoring a ticagrelor monotherapy strategy over standard DAPT among non-diabetic (2.3% vs. 5.3%; HR, 0.43; 95% CI, 0.26–0.70) but not among diabetic subjects (4.1% vs. 4.7%; HR, 0.87; 95% CI, 0.45–1.68; *p* for interaction = 0.09)

ACS, acute coronary syndrome; ATS, atherosclerotic vascular disease; CCS, chronic coronary syndrome; CI, confidence interval; CV, cardiovascular; DAPT, dual antiplatelet therapy; DES, drug-eluting stent; MACE, major adverse cardiovascular events; MI, myocardial infarction; PCI, percutaneous intervention; HR, hazard ratio; RR, rate ratio; SAPT, single antiplatelet therapy.

In the GLOBAL LEADERS trial (*n* = 15,968, 26% diabetics, 8% insulin-treated), it was found that ticagrelor monotherapy for 2 years following PCI did not demonstrate superiority over the standard DAPT in preventing the primary composite ischemic endpoint or major bleeding, even in diabetic patients (34). In the TWILIGHT trial (*n* = 5,119, 37% diabetics, 10% insulin-treated), it was found that high-risk patients undergoing PCI using a ticagrelor SAPT for 12 months after 3 months of DAPT experienced fewer major bleeding events compared with those who underwent standard DAPT, with no excess in ischemic events reported in both diabetic and non-diabetic patients (35, 36).

Other recent RCTs explored a P2Y_12_ inhibitor SAPT in high-risk patients with an ACS with conflicting results. The TICO trial (*n* = 3,056, 27% diabetics) showed that a ticagrelor SAPT after a 3-month DAPT was associated with a modest, but significant, reduction in the primary composite endpoint of major bleeding and MACE, a difference mainly driven by a lower risk for major bleeding (37). The treatment effect was consistent among patients with and without diabetes (37). In the STOPDAPT2 ACS trial (*n* = 4,169, 30% diabetics), it was found that a clopidogrel SAPT after 1–2 months of DAPT fell short of demonstrating non-inferiority compared with the standard 12-month DAPT with regard to the primary composite endpoint of CV death, MI, any stroke, definite ST, and bleeding events, with a numerical increase in CV events despite significant reductions in major bleeding events (38). The treatment effect was consistent among patients with and without diabetes (38). Overall, these RCTs indicate that shorter durations of DAPT, followed by P2Y_12_ SAPT, might reduce major bleeding events without significantly affecting MACE (39). Notably, diabetic patients appeared to benefit from P2Y_12_ inhibitor SAPT strategies, with reduced major bleeding events and potential reductions in MACE.

Two recent randomized trials have investigated the clinical benefits of a P2Y_12_ inhibitor SAPT without DAPT (40) or within a 1-month DAPT (41) following PCI in patients with an ACS or those with high bleeding risk. When compared with a conventional DAPT with aspirin and prasugrel following PCI, low-dose prasugrel-based SAPT was found to be non-inferior in reducing CAD MACE but not effective in reducing major bleeding events, at 1-month follow-up in the STOPDAPT-3 trial (*n* = 6,002, 40% diabetics) (40). The treatment effect was consistent among patients with and without diabetes (42).

In the T-PASS study (*n* = 2,850, 30% diabetics), stopping aspirin within 1 month (median of 16 days) and transitioning to a ticagrelor SAPT among patients with ACS who underwent PCI were found to be both non-inferior and superior to a 12-month DAPT in terms of the 1-year composite outcome of death, MI, ST, stroke, and major bleeding, mainly driven by a significant reduction in major bleeding events (43). Significant treatment-by-diabetic status interaction regarding the occurrence of the composite ischemic endpoint favored a ticagrelor monotherapy strategy over the standard DAPT among non-diabetic, but not in diabetic subjects (43).

Long-term SAPT with a P2Y_12_ inhibitor may be appropriate, as shown by a recent meta-analysis that revealed lower risks of CV death, MI, and stroke compared with aspirin monotherapy in patients with CAD with similar major bleeding risk (44). A P2Y_12_ inhibitor SAPT following a short DAPT course may therefore represent a promising alternative to a standard DAPT after PCI with a new-generation DES among diabetic patients (45), but these findings warrant confirmation from dedicated RCTs.

### Glycoprotein IIb/IIIa receptor inhibitors

Intravenous GP receptor inhibitors were found to reduce 30-day mortality in patients with ACS, especially in diabetic patients undergoing PCI, but this benefit had been more prominent before the current practice of routine potent P2Y_12_ receptor inhibitor use (46). In today's era of oral P2Y_12_ receptor inhibitors, intravenous GP IIb/IIIa receptor inhibitors should be considered only in patients with ACS for bailout situations arising during PCI, such as no-reflow or thrombotic complications (40).

## Long-term antithrombotic therapies following PCI in diabetic patients

### Antiplatelet therapy

Prolonged DAPT durations have been advocated to improve stent-related outcomes in patients with high ischemic and low bleeding risk (Table 2).

**Table 2:** Summary of major randomized clinical trials and subgroup analyses studying prolonged double antiplatelet therapy in patients with diabetes after PCI.

Study	Year	Patients (diabetic %)	Intervention	Follow-up	Outcomes
DAPT	2014	9,961 patients (diabetics, 31%)	Aspirin and PCI with DES (newer-generation DES, 60%) treated with DAPT combining aspirin and thienopyridine (clopidogrel, 65%; prasugrel, 35%), for 12 months, receiving thienopyridine vs. placebo during another 18 months in addition to aspirin	30 months	• Prolonged DAPT with thienopyridine was associated with significantly lower rates of stent thrombosis (0.4% vs. 1.4%; HR, 0.29; 95% CI, 0.17–0.48; *p* < 0.001), MACE (4.3% vs. 5.9%; HR, 0.71; 95% CI, 0.59–0.85; *p* < 0.001), and MI (2.1% vs. 4.1%; HR, 0.47; *p* < 0.001) compared with placebo • There was a significant increase in moderate or severe bleeding events with continued thienopyridine (2.5% vs. 1.6%, *p* = 0.001) • Diabetic patients derived less clinical benefits from a prolonged DAPT strategy post PCI with respect to stent thrombosis (*p* for interaction = 0.08) and MACE (*p* for interaction = 0.01) compared with non-diabetic individuals
CHARISMA	2006	15,603 patients (diabetics, 42%) with established atherosclerotic disease or multiple CV risk factors	Long-term administration of DAPT with aspirin (75–162 mg/day) and clopidogrel (75 mg/day) in comparison with aspirin alone	28 months	• No significant difference between patients treated with aspirin and clopidogrel when compared with those receiving aspirin alone with respect to the primary composite of CV death, MI, or stroke (6.8% vs. 7.3%; RR, 0.93; 95% CI, 0.83–1.05; *p* = 0.22) and severe bleeding events (1.7% vs. 1.3%; RR, 1.25; 95% CI, 0.97–1.61; *p* = 0.09) • In the large subgroup of symptomatic patients with clinically evident atherothrombosis (*n* = 12,153), DAPT with aspirin and clopidogrel was found superior to aspirin alone in reducing the primary combined endpoint (6.9% vs. 7.9%; RR, 0.88; 95% CI, 0.77–0.998; *p* = 0.046) • No significant difference between treatment groups among asymptomatic patients (6.6% vs. 5.5%; *p* = 0.20; *p* for interaction = 0.045) • In the large subgroup of diabetic patients (*n* = 6,556), no significant interaction between treatment effect and diabetic status was observed
THEMIS	2020	19,220 patients with CCS and type 2 diabetes, without prior MI or stroke	Ticagrelor (90 mg twice daily, 74%; 60 mg twice daily, 26%) or placebo, in addition to aspirin	39.9 months	• Incidence of the primary combined endpoint of CV death, MI, or stroke was significantly lower among diabetic patients treated with ticagrelor than among those receiving placebo (7.7% vs. 8.5%; HR, 0.90; 95% CI, 0.81–0.99; *p* = 0.04) • Incidence of major bleeding events (2.2% vs. 1.0%; HR, 2.32; 95% CI, 1.82–2.94; *p* < 0.001) and intracranial hemorrhage (0.7% vs. 0.5%; HR, 1.71; 95% CI, 1.18–2.48; *p* = 0.005) were significantly higher in ticagrelor-treated patients
PEGASUS-TIMI 54	2015	21,162 patients (diabetics, 32%) with prior MI 1–3 years earlier	Ticagrelor 90 mg twice daily, ticagrelor 60 mg twice daily, or placebo in addition to low-dose aspirin	33 months	• Both ticagrelor doses were shown to significantly reduce the rates of the composite of CV death, MI, or stroke (90 mg, 7.85%; 60 mg, 7.77%) compared with placebo (9.04%; HR for ticagrelor 90 mg vs. placebo, 0.85; 95% CI, 0.75–0.96; *p* = 0.008; HR for ticagrelor 60 mg vs. placebo, 0.84; 95% CI, 0.74–0.95; *p* = 0.004) • Rates of major bleeding were significantly higher with ticagrelor (90 mg, 2.60%; 60 mg, 2.30%) than with placebo (1.06%; *p* < 0.001 for each ticagrelor dose vs. placebo) • No significant differences were found with respect to intracranial hemorrhage or fatal bleeding among the three treatment groups

ACS, acute coronary syndrome; ATS, atherosclerotic vascular disease; CCS, chronic coronary syndrome; CI, confidence interval; CV, cardiovascular; DAPT, dual antiplatelet therapy; DES, drug-eluting stent; MACE, major adverse cardiovascular events; MI, myocardial infarction; PCI, percutaneous intervention; HR, hazard ratio; RR, rate ratio; SAPT, single antiplatelet therapy.

In the DAPT trial (*n* = 9,961, 31% diabetics), it was found that individuals who had undergone PCI with a new-generation DES and received prolonged DAPT with thienopyridine for 30 months had lower rates of ST, MACE, and MI compared with those in the placebo group (47). However, there was a notable increase in moderate to severe bleeding events associated with continued thienopyridine use. Interestingly, diabetic patients appeared to derive less benefit from prolonged DAPT (47).

Diabetic patients face a higher long-term risk of atherothrombotic events and have been studied for more intensive antithrombotic therapy (11). The THEMIS trial (*n* = 19,220), focusing on diabetic patients with CCS, found that ticagrelor added to aspirin, when compared with aspirin alone, reduced the primary composite and clopidogrel endpoint of CV death, MI, or stroke but came with a higher risk of major bleeding and intracranial hemorrhage (48). A subgroup analysis showed similar trends in diabetic patients undergoing PCI (49). In the PEGASUS-TIMI 54 trial (*n* = 21,162, 32% diabetics), which included patients with a history of MI, it was found that ticagrelor, added to low-dose aspirin, at both 60 and 90 mg doses reduced the composite of CV death, MI, or stroke in these patients, compared with the placebo group of patients but carried a higher risk of major bleeding events, with the 60 mg dose appearing to have a more favorable benefit-risk profile than the 90 mg dose in both diabetic and non-diabetic patients (50).

Overall, long-term DAPT with aspirin and ticagrelor may be considered for diabetic patients with a history of MI or prior PCI who have tolerated antiplatelet therapy and are deemed at high ischemic (e.g., a complex left main stem, a two-stent bifurcation, a suboptimal stenting result, and a prior stent thrombosis, previously known as CYP2C19*2/*3 polymorphisms) (51) and low bleeding risks, as proposed by the Academic Research Consortium for high bleeding risk (ARC-HBR) criteria (52).

### Direct oral anticoagulants

The potential clinical benefits of an intensified antithrombotic regimen with direct oral anticoagulants added to conventional antiplatelet therapy among patients with ACS or those who undergo PCI have been investigated in several RCTs. The ATLAS ACS 2-TIMI 51 trial (*n* = 15,526, 32% diabetics) demonstrated that adding low-dose rivaroxaban (2.5 or 5 mg) to DAPT in patients with a recent ACS reduced the risk of CV death, MI, or stroke compared with the placebo group of patients (53). However, it significantly increased the risk of major bleeding events, with less benefit observed in diabetic patients (53). In the GEMINI-ACS-1 trial (*n* = 3,037, 30% diabetics), it was found that combining low-dose rivaroxaban with a P2Y_12_ inhibitor, alongside aspirin, in patients with ACS undergoing PCI was deemed safe, with similar major bleeding rates, without any significant difference in patients with and without diabetes (54). However, larger trials are needed to validate this approach for bleeding and ischemic endpoints before recommending this treatment strategy.

In the COMPASS trial (*n* = 27,395, 38% diabetics), it was found that the combination of aspirin and low-dose rivaroxaban among patients with stable atherosclerotic CV disease reduced the risk of CV death, stroke, or MI compared with aspirin alone, but it increased the risk of major bleeding events (55). Rivaroxaban alone did not provide additional CV benefits and was associated with more major bleeding events (55). The treatment effect was consistent in diabetic patients. A decision to use this combination therapy should consider patient characteristics, including bleeding risk. Current guidelines regarding both acute and chronic coronary syndromes recommend that adding a second antithrombotic agent such as rivaroxan 2.5 mg to aspirin for extended long-term secondary prevention should be considered in patients with high ischemic risk and those without high bleeding risks (40, 51). As most patients enrolled in COMPASS had vascular disease and the benefit of the combination seemed more pronounced in this population, low-dose rivaroxaban, in addition to aspirin, is commonly administered to patients with advanced peripheral vascular disease.

## Other secondary prevention therapies

### Lipid-lowering therapies

Dyslipidemia is a major CV risk factor frequently encountered in diabetic patients (33). Compared with their non-diabetic counterparts, diabetic patients with dyslipidemia have higher levels of atherogenic triglyceride-rich particles due to hyperinsulinemia and glycosylation of small dense low-density lipoprotein cholesterol (LDL-c) particles (41). Lipid-lowering therapy is strongly recommended as a preventive therapy in diabetic patients following PCI (56). Overall, the diabetic population, who generally represent 20%–40% of the trial population in secondary prevention, tends to benefit more than non-diabetic patients from LDL-c-lowering therapies, with a larger absolute risk reduction and lower treatment numbers.

### Statins

Statins reduce the risk of MACE by 21% for each 1 mmol/L reduction of LDL-c including all-cause and CV mortality (57). The risks of MI, coronary revascularization, and stroke are also significantly decreased with statin in diabetic patients, regardless of preexisting vascular disease (57). The IBIS-4 study elucidated the mechanisms as high-intensity rosuvastatin was associated with a regression of coronary atherosclerosis in non-infarct-related arteries following ST-elevation MI (58). Current guidelines recommend high-intensity statin therapy in high-risk patients regardless of LDL-c values (56). These recommendations are applicable to diabetic patients who undergo PCI (59).

### Ezetimibe

The benefits of lowering LDL-c on clinical outcomes are also reported in diabetic patients with vascular disease with non-statin agents. The addition of ezetimibe significantly reduces LDL-c levels up to a significant 15% relative reduction and a 5.5% absolute risk reduction of the primary endpoint of MACE, a composite of CV death, major coronary events, or stroke (60). Because the absolute risk is higher in diabetics, and even higher in those with concomitant polyvascular disease, the addition of ezetimibe provides greater absolute risk reductions (61). Current guidelines recommend the addition of ezetimibe on top of statin therapy in high-risk patients who do not reach LDL-c targets (56). These recommendations are applicable to diabetic patients who undergo PCI (59).

### PCSK9 inhibitors

Two proprotein convertase subtilisin–kexin type 9 (PCSK9) inhibitors, evolocumab and alirocumab, were studied in secondary prevention trials with a significant proportion of diabetic patients. In the FOURIER trial (*n* = 27,564, 40% diabetics), it was found that evolocumab reduced LDL-c levels by 57% and lowered MACE in diabetic patients, without increasing the risk of new-onset diabetes, compared with the placebo group of patients (62). Similarly, in the ODYSSEY OUTCOMES trial (*n* = 5,444, 29% diabetics), it was found that alirocumab, on top of maximally tolerated lipid-lowering therapy, reduced LDL-c levels by 64% at 4 months and reduced MACE rates, without increasing the risk of new-onset diabetes (63), compared with the placebo group. Current guidelines recommend the addition of PCSK9 inhibitors on top of statin therapy and ezetimibe in high-risk patients who do not reach the LDL-c targets (56). These recommendations are applicable to diabetic patients who undergo PCI (59).

### Icosapent ethyl

High-dose marine omega-3 supplementation with icosapent ethyl was studied as an alternative to improve clinical outcomes in high-risk patients (64), such as in the REDUCE-IT trial (*n* = 8,179, 58% diabetics), which involved patients with high triglyceride levels. This supplementation led to a 34% MACE compared with a placebo (65). Similar results have been shown in the JELIS trial (*n* = 18,645, 16% diabetics), with a 19% relative reduction in major coronary events in Japanese patients with high levels of total cholesterol (66). Accordingly, current guidelines recommend the use of marine omega-3 supplementation for the treatment of hypertriglyceridemia in patients with an eligible range of triglycerides between 1.5 and 5.6 mmol/L (56). These recommendations are applicable to diabetic patients undergoing PCI (59).

### Other therapies

Inclisiran is a small interfering RNA that reduces the production of PCSK9 (41). Its effect on lowering LDL-c levels has been shown to be similar to that of monoclonal antibodies against PCSK9 (41). Its efficacy in reducing MACE is being currently investigated in the ORION-4 trial (NCT03705234). Bempedoic acid is another LDL-c-lowering compound that inhibits the peroxisome proliferator-activated receptor, used as an additional therapy or for patients who are intolerant to statins (41), associated with a lower risk of MACE (composite of death from CV causes, non-fatal MI, non-fatal stroke, or coronary revascularization) compared with placebo in the CLEAR Outcomes trial (*n* = 13,970, 46% diabetics) (67). A subgroup analysis suggested a particular benefit for diabetic patients, with postulated mechanisms such as apoB lipoprotein and high-sensitivity C-reactive protein reduction (41).

### Antidiabetic drugs

Among antidiabetic drugs, only GLP1-receptor agonists (GLP1-RA) and SGLT2 inhibitors (SGLT2i) have been shown to significantly reduce the risk of MACE in diabetic patients (68).

SGLT2i has shown benefits in reducing MACE in patients with type 2 diabetes (69), especially the risk of CV death in diabetic patients with prior MI (70), with a MACE reduction up to 16% for dapagliflozin (71). SGLT2i may improve outcomes among survivors of MI by attenuating neurohormonal activation, cardiomyocyte necrosis, and reperfusion injury (70). GLP1-RA has also demonstrated benefits in reducing MACE, especially in patients with established CV disease (68). It may reduce infarct size and improve left ventricular ejection fraction in patients with an acute MI undergoing PCI (72). The current guidelines strongly recommend SGLT2i along with GLP1-RA in diabetic patients with established CV disease (59). However, the CV benefits of these glucose-lowering drugs with proven efficacy remain not fully understood.

In addition, it was found that SGLT2i reduced heart failure–related endpoints and the progression of kidney disease (59, 73, 74). However, the recent EMPACT-MI and DAPA-MI trials, which explored the use of SGLT2 inhibitors following an acute MI, failed to demonstrate a reduction in first hospitalization for heart failure (HF) or death from any cause (75) nor in the composite of CV death or hospitalization for HF (76) compared with placebo. Therefore, iSGLT2 is recommended to lower the risk of heart failure hospitalization in diabetic patients who have, or are at risk of, heart failure or chronic kidney disease (59).

## Conclusion

The current recommendations regarding antiplatelet and lipid-lowering treatments do not differ between diabetics and non-diabetics after they undergo PCI or experience an ACS. The use of SGLT2i and GLP1-RA in diabetics with established CV disease is strongly recommended to reduce MACE.
